# Evaluation of Chest CT Scan as a Screening and Diagnostic Tool in Trauma Patients with Coronavirus Disease 2019 (COVID-19): A Cross-Sectional Study

**DOI:** 10.1155/2021/4188178

**Published:** 2021-06-28

**Authors:** Hossein Abdolrahimzadeh Fard, Salahaddin Mahmudi-Azer, Sepideh Sefidbakht, Pooya Iranpour, Shahram Bolandparvaz, Hamid Reza Abbasi, Shahram Paydar, Golnar Sabetian, Mohamad Mahdi Mahmoudi, Masoume Zare, Leila Shayan, Maryam Salimi

**Affiliations:** ^1^Trauma Research Center, Department of Surgery, Shahid Rajaee (Emtiaz) Trauma Hospital, Shiraz University of Medical Sciences, Shiraz, Iran; ^2^Pulmonary Research Group, Department of Medicine, University of Alberta, Edmonton, Alberta, Canada; ^3^Medical Imaging Research Center, Department of Radiology, Shiraz University of Medical Sciences, Shiraz, Iran; ^4^Education Development and Research Center, Shiraz University of Medical Sciences, Shiraz, Iran; ^5^Shiraz Anesthesiology and Critical Care Research Center, Shiraz University of Medical Sciences, Shiraz, Iran; ^6^Student Research Committee, Shiraz University of Medical Sciences, Shiraz, Iran

## Abstract

**Background:**

The lack of enough medical evidence about COVID-19 regarding optimal prevention, diagnosis, and treatment contributes negatively to the rapid increase in the number of cases globally. A chest computerized tomography (CT) scan has been introduced as the most sensitive diagnostic method. Therefore, this research aimed to examine and evaluate the chest CT  scan as a screening measure of COVID-19 in trauma patients.

**Methods:**

This cross-sectional study was conducted in Rajaee Hospital in Shiraz from February to May 2020. All patients underwent unenhanced CT with a 16-slice CT scanner. The CT scans were evaluated in a blinded manner, and the main CT scan features were described and classified into four groups according to RSNA recommendation. Subsequently, the first two Radiological Society of North America (RSNA) categories with the highest probability of COVID-19 pneumonia (i.e., typical and indeterminate) were merged into the “positive CT scan group” and those with radiologic features with the least probability of COVID-19 pneumonia into “negative CT scan group.”

**Results:**

Chest CT scan had a sensitivity of 68%, specificity of 56%, positive predictive value of 34.8%, negative predictive value of 83.7%, and accuracy of 59.3% in detecting COVID-19 among trauma patients. Moreover, for the diagnosis of COVID-19 by CT scan in asymptomatic individuals, a sensitivity of 100%, specificity of 66.7%, and negative predictive value of 100% were obtained (*p* value: 0.05).

**Conclusion:**

Findings of the study indicated that the CT scan's sensitivity and specificity is less effective in diagnosing trauma patients with COVID-19 compared with nontraumatic people.

## 1. Background

In March 2020, the World Health Organization (WHO) declared COVID-19 a pandemic. Although it has been nine months into the novel coronavirus disease 2019 (COVID-19) pandemic, the high number of daily cases still remains problematic [1, 2]. By September 16, 2020, almost 30 million cases of COVID-19 had been confirmed by laboratory tests, including about 1 million deaths [3, 4]. Mortality rates were reported as 8% in the south of Iran, the country that is considered one of the most major focal points of the world's disease. It has also had a substantial effect on mental health [5–7]. Although its worldwide mortality rate is less than 4% today, the lack of definitive treatment [8] makes the diagnosis of infected people to break the disease chain the primary method of dealing with the disease. The foregoing issue necessitates a faster diagnostic method of suspected individuals with definitive diagnostic tests. A large number of asymptomatic carriers have led to greater efforts to identify asymptomatic patients. Concerning the low sensitivity and time-consuming nature of the current serological tests, efforts have been focused on using faster and more sensitive diagnostic tests [9]. The use of a chest CT scan has been found as a promising screening method in asymptomatic patients [10]. Moreover, by improving the definitive diagnostic methods and comparing the findings of chest CT scans in the definitely infected patients, it became clear that chest CT scans can be used with high sensitivity in diagnosing the patients [11]. Even though the Radiology Society of North America (RSNA) has standardized chest CT scan reports, its low negative predictive value has overshadowed the use of CT scans as a convenient screening method [12]. Likewise, chest CT scan is not recommended as a screening tool for asymptomatic patients not only due to the resources required and unnecessary radiation to patients but also because of the low predictive value. Furthermore, while chest CT has a relatively high sensitivity, the specificity is relatively low, with COVID-19 pneumonia exhibiting overlapping features with numerous other diseases; CT cannot be used as a form of diagnosis.

Trauma centers, due to the nature of the patients referred to them and the time constraints they face due to the priority of treating life-threatening injuries, need to perform treatments that cause close contact with the patient and are particularly challenging when dealing with potential COVID-19 patients. Given the extensive use of CT scan as the gold standard diagnostic method in most trauma centers, its availability, and short time to perform, this retrospective study evaluates the sensitivity and specificity of chest CT scan in the diagnosis of COVID-19 in trauma patients. This study investigated the effect of trauma on the clinical findings of CT scans in COVID-19 based on RSNA classification. We also aimed to evaluate the utility of chest CT to help triage trauma patients for potential presence of COVID-19 infection.

## 2. Methods

### 2.1. Patients and Data Source of RT-PCR Results

This cross-sectional study was conducted in Rajaee Hospital in Shiraz, the only dedicated trauma center in Iran with an annual referral rate of 15,000 to 20,000 patients, from February to May 2020. Trauma patients who needed to undertake a chest CT scan for their trauma were included in the study. These patients have been checked for COVID-19 at the time of hospitalization, based on the Quality Improvement Committee's approved protocol. Data regarding the vital signs, submitted tests, and COVID-19-related history have been completed from the medical record system. Patients who did not have a CT scan and RT-PCR test at the time of their arrival or the interval between them was more than 24 hours were excluded from the study. Those who had lung-related underlying disease such as chronic obstructive pulmonary disease (COPD) and lung cancer were also excluded. Clinical symptoms and epidemiological risk factors that were associated with COVID-19 and the underlying diseases were obtained.

This study used a correlational design to examine the relationship between chest CT scan finding and RT-PCR result as the golden standard for definite COVID-19 diagnosis. Patients with a negative RT-PCR test have been rechecked using the lower respiratory tract after four days.

### 2.2. Chest CT Protocol

All CT scans were obtained with one CT system. All patients underwent unenhanced CT with a 16-slice CT scanner (CT emotion 16, Siemens Healthcare, Erlangen, Germany). Given the probability of COVID-19, all necessary protective measures have been taken according to the Iranian Ministry of Health guidelines [13].

### 2.3. Image Analysis

Two radiologists with Iranian Board Certification (SS and PI), who were blinded to RT-PCR result and COVID-19-related symptoms, reported all CT scans by consensus. They described main CT scan features (ground-glass opacity (GGO), consolidation, bronchial distortion, reticular line, crazy paving sign, atoll sign, cavity, rib fracture, pneumothorax, hemothorax, diaphragm injury, and shape and pattern of the lesions) and classified the patients into four groups according to RSNA recommendation [14] (Table 1). A chest CT  scan is considered to be the most available diagnostic modality in the setting of chest trauma. Therefore, we hypothesized the possibility of utilizing it as an initial screening test for possible identification of the concomitant COVID-19 pneumonia in the trauma cases and also for the proper allocation of the patients to COVID-19 and non-COVID-19 wards before the RT-PCR tests were processed. Therefore, we merged the first two RSNA categories with the highest probability of COVID-19 pneumonia (i.e., typical and indeterminate) into the “positive CT scan group” and those with radiologic features with the least probability of COVID-19 pneumonia into the “negative CT scan group.”

### 2.4. Statistical Analysis

The means and standard deviations were calculated for quantitative variables, and count and percentage were calculated for qualitative variables. A comparison between the two groups was performed using the chi-square test or Fisher's exact test. Moreover, sensitivity and specificity have been calculated to determine the correct diagnosis of different protocols. SPSS version 16 (IBM, United States) was used for statistical analysis. The significance level was considered at 0.05.

## 3. Results

**Figure 1:**
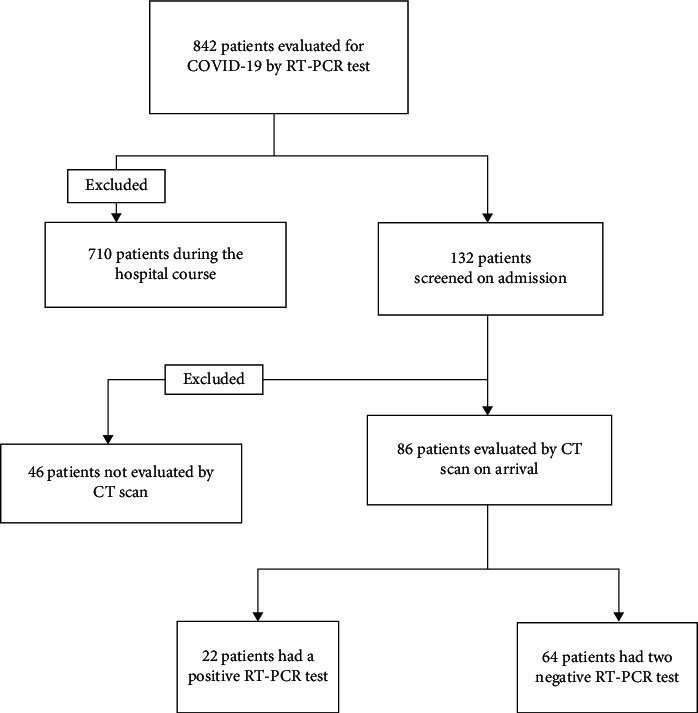
Pathway design of the study.

Of 842 PCR tests performed, 132 cases were screened on admission according to the hospital guidelines with 30 positive results. According to the following chart, 86 patients (male: 82.6%; female: 17.4%; mean age: 40.5 ± 20.4) underwent a chest CT scan according to the trauma mechanism, and the surgical guidelines were included in the study (Figure 1).

Typical and indeterminate reports were classified as positive CT scans for COVID-19, while atypical and normal CT scans were reported as a negative group. According to the outcomes of CT scan, 44 patients (male: 85.7%; female: 14.3%) had typical and indeterminate findings for COVID-19 and 42 patients (male: 79.5%; female: 20.5%) had atypical or normal CT finding. The analysis revealed no significant statistical difference between the two groups on age, sex, and history of underlying diseases (Table 2). Comparison of race, clinical symptoms, and epidemiological risk factors associated with COVID-19 between positive and negative groups showed that the Iranian race (97.7%, 81%, PV: 0.014), incidence of acute respiratory symptoms (63.9%, 36.1%, PV: 0.037), and history of dyspnoea (38.6%, 16.7%, PV: 0.02) had significant differences in the existence between the two types of CT scan groups. In correlation with the RT-PCR result, 31.8% definite patients had a negative chest CT scan, whereas 100% of them were likely to be infected by COVID-19 and 28.3% were highly suspicious for COVID-19. In the patients with the negative RT-PCR result, 43.7% had positive chest CT scan findings, of which 75% could be infected by COVID-19.

Patients were divided into four groups according to different RSNA categories. According to RSNA classification, the chest CT scans were reported as typical (26.7%), indeterminate (24.4%), atypical (29.1%), and normal (19.8%). In correlation with RT-PCR result in definite COVID-19 group, incidence of RSNA type (T/I/A/N) was 31.8%, 36.4%, 27.3%, and 4.5%. In a typical group, 69.6% had negative RT-PCR and 30.4% had positive RT-PCR results. In patients with a report of normal CT scan, 5.9% had a positive RT-PCR result.

There was a significant difference between the four groups of RSNA (T, I, A, N) regarding the mean of age (48.7, 36.8, 43.6, and 30.3; PV: 0.023), Iranian nationality (95.7, 100, 86.2, and 77.8; PV: 0.023), and incidence of acute respiratory symptoms (52.2, 45, 87, and 44.4; PV: 0.012). None of the respiratory symptoms were significantly different between the four groups.

Based on the RT-PCR test results, patients were divided into two groups of definite and noninfected patients, and based on the diagnostic and nondiagnostic type of CT scan and RSNA recommendation classes, different features, sensitivity, specificity, positive predictive value, negative predictive value, and accuracy of them were assessed for a diagnosis of definite patients. Incidence of different common COVID-19 features in chest CT scan was investigated in these two groups (Table 3).

The sensitivity and specificity of the positive CT group, typical RSNA type, and different features of the CT scan report were calculated as shown in Table 4.

Positive chest CT scans for COVID-19 were compared between two groups according to RT-PCR results. Fifteen patients with positive RT-PCR test and 28 patients with negative RT-PCR results had positive chest CT scans features for COVID-19. So, a chest CT scan had a sensitivity of 68%, specificity of 56%, positive predictive value of 34.8%, negative predictive value of 83.7%, and accuracy of 59.3% in detecting COVID-19 among trauma patients.

Evaluation of 23 asymptomatic patients (two with positive RT-PCR and 21 negative RT-PCR results) showed that all definitively infected patients have a positive CT scan for the disease and only seven noninfected patients have a positive CT scan. Statistical analysis showed a sensitivity of 100% and specificity of 66.7% and negative predictive value of 100% for the diagnosis of COVID-19 by CT scan in asymptomatic individuals.

## 4. Discussion

The early diagnosis of COVID-19 plays an important role in monitoring the disease effectively. According to the initial definition provided by the World Health Organization, patients with suspected clinical symptoms should be evaluated by RT-PCR testing for COVID-19 [15]. Related antibodies need to be evaluated for definitive COVID-19, which is conducted by evaluating the presence of the virus in different ways. The results of these methods are related to different factors such as the time of exposure to the virus and the onset of symptoms, which are effective in positive serological tests [9, 16]. The diagnosis of asymptomatic patients and patients with mild symptoms, which includes a high percentage of patients [17], is also an important goal of healthcare organizations. Taken into consideration the low sensitivity of definitive diagnostic tests (RT-PCR) [9, 18], other diagnostic methods are used to accompany them [19–21]. Typical chest CT scan findings in people with definite COVID-19 pneumonia (Table 1) are one of the most commonly used methods. This study investigated the effectiveness of RSNA classification and its features as a diagnostic tool for COVID-19 in trauma patients. Due to the presence of similar manifestations of lung contusion in chest CT scan and also the possibility of simultaneous occurrence of these two pathologies, their interactions on CT scan findings must be evaluated in trauma patients [22]. Evaluation of chest CT scan findings based on RSNA classification showed the lower sensitivity and specificity among the trauma patients. This outcome can be related to the prevalence rate of the disease, low sensitivity of the RT-PCR diagnostic test, and the weakness of the chest CT scan findings, which attributed to infected nontraumatic patients for traumatic individuals.

Given the high prevalence of the disease in the area where the study was conducted and the acceptable sensitivity of the diagnostic tests, the findings of the chest CT scan for COVID-19 as a diagnostic tool might differ among trauma patients.

Patients were divided into positive and negative groups based on their chest CT scan findings, regardless of RT-PCR serological test results. Evaluation of clinical symptoms related to COVID-19, systemic inflammatory response syndrome (SIRS) index and lymphocyte-to-neutrophil ratio (NLR) as a marker of systemic inflammatory responses, factors related to patients' trauma including primary vital signs as well as injury severity score (ISS) and abbreviated injury score (AIS), patient's hospital course, and prognosis of patients between the two groups was performed. The evaluation showed that patients were not significantly different in terms of age, COVID-19-related symptoms (except for respiratory distress), and epidemiological and underlying disease history. The evaluation of inflammatory and immune system stimulation factors as well as criteria related to the severity of trauma did not show a significant difference between the two groups. With the exception of race and respiratory distress, factors related to COVID-19 and inflammatory system and trauma severity do not affect the probability of positive chest CT scan according to the criteria provided by RSNA.

The outcomes of this research are not in line with studies investigating nontraumatic individuals [23]. Trauma and COVID-19 both stimulate the immune system. However, the degree of stimulation of the inflammatory system depends on the degree of trauma severity and also the inflammatory stage of COVID-19. It is hypothesized that if the criteria set by RSNA were appropriate for the evaluation of COVID-19 traumatic individuals, a significant difference should be observed between the two groups.

Evaluation of common radiological features based on RSNA classification between the two groups of definitive and noninfected patients showed that the presence of peripheral, bilateral, round, and diffuse GGO as the most specific radiological features in nontraumatic individuals [24] is not similar in traumatic patients. Although multiple bilateral GGO was the most sensitive in lesions (45%) according to the location in trauma patients, the specificity of multiple unilateral lesions was higher (96%). Moreover, the peripheral lesions were more sensitive and the central lesions had the highest specificity (93%). In terms of lesion shape, the highest sensitivity was related to irregular lesions (40%) and the highest specificity was related to round lesions (96%). Therefore, multiple unilateral or central GGO in trauma patients was the most specific type of GGO in trauma patients who had definite COVID-19. Regarding consolidation, peripheral round lesions had the most diagnostic features in nontraumatic patients, but central lesions (98%) with mixed round and irregular shapes had the highest diagnostic features in traumatic patients with COVID-19.

Despite the previously mentioned data, due to the possibility of co-occurrence of COVID-19 and pulmonary contusion in both groups, the findings of this research should be treated with caution and future studies should collect information about trauma patients in the prepandemic period of COVID-19 to determine specific lung contusion lesions.

Therefore, it seems that, for the detection of COVID-19 in trauma patients, it is necessary to provide another classification for a chest CT scan. This requires further studies with a larger population and also the use of more sensitive definitive diagnostic tests.

In the evaluation of 17 patients with rib fractures in both definite and noninfected patients, it was found that rib fracture in 100% of definite patients and 71% noninfected patients leads to damage to the pulmonary parenchyma and pleura. Also, patients with less ISS in a definite group develop more symptoms of regional injury in the thoracic cavity (AIS). This indicates that the presence of underlying pathology due to COVID-19 in traumatic individuals causes the lung parenchyma to be prone to injury and rupture. Moreover, it seems that the ISS/thoracic cavity AIS ratio may contribute to the possibility of underlying pathology in traumatized individuals. However, due to the small number of participants, it was not possible to statistically evaluate this finding further. There were some limitations in our study. It is possible that patients could not remember the associated symptom of COVID-19 or the history of contact with a suspicious person. Further multicentric studies with a large sample size were essential to find the complete disadvantages of using the routine criteria in the trauma patient. Overall, the results of this study can be the basis for further studies to introduce a new triage system and criteria in trauma patients.

## 5. Conclusion

The results show that RSNA criteria for COVID-19 were not efficient in trauma patients. Therefore, due to the high use of CT scans in trauma patients, it is recommended to create appropriate CT scan criteria for trauma patients. This method could diagnose the disease timely and contribute positively to the termination of the transmission chain. It would also reduce the incidence of treatment and effective resource management.

## Figures and Tables

**Table 1:** RSNA criteria for COVID-19 pneumonia (adopted from reference [14]).

COVID-19 RSNA classification	Rationale	Chest CT scan findings
Typical appearance	Commonly reported imaging feature of greater specificity for COVID-19	(i) Peripheral and bilateral GGO with/without consolidation
		(ii) Multifocal GGO with/without crazy paving sign
		(iii) Reverse halo sign

Indeterminate feature	Nonspecific image for COVID-19	(i) Absence of typical feature
		(ii) Presence of multifocal, diffuse, prehilar, or unilateral GGO with/without nonround, nonperipheral consolidation
		(iii) Few very small GGO, nonround and nonperipheral distribution

Atypical appearance	Uncommonly or not reported feature of COVID-19	(i) Absence of typical and atypical features
		(ii) Presence of single lobar or segmental consolidation, discrete small nodules, lung cavitation, and smooth interlobular septal thickening with pleural effusion

Normal for pneumonia	No features of pneumonia	(iii) No CT features to suggest the pneumonia

CT: computed tomography; COVID-19: coronavirus disease 2019; GGO: ground-glass opacity.

**Table 2:** Comparison of COVID-19-related criteria between positive and negative CT scan groups.

Variables	Chest CT scan +ve (%) *n* = 44	Chest CT scan −ve (%) *n* = 42	*p* value
*Nationality*			
Iranian	97.7	81	0.014
Non-Iranian	2.3	19	

*Positive COVID-19-related history*			
Contact	6.8	14.3	0.3
Fever	27.1	38.1	0.2
Cough	13.6	11.9	0.53
Respiratory distress	38.6	16.7	0.02
Neurological symptoms	15.9	4.8	0.08
Dermatological symptoms	0	0	—
Abdominal pain	2.3	2.4	1
Anorexia	6.8	2.4	0.61

*Positive past medical history*			
Cancer	0	2.4	0.48
Diabetes mellitus	9.1	7.1	1
Chronic anemia	0	2.4	0.48
Cardiac disease	9.1	11.9	0.73
Chronic renal failure	2.3	0	1
Asthma	2.3	0	1
COPD	2.3	2.4	1
HTN	9.1	4.8	0.67

*Hospital course*			
Intubation need	52.3	35.7	0.09
ICU admission	77.3	61.9	0.09
ARDS incidence	39	41.7	0.75
Mortality	25	9.5	0.53

*Patient classification*			
Suspicious (WHO)	2.3	4.8	0.61
Possible (ECDC)	79.5	66.7	0.13
Asymptomatic	20.5	33.3	0.2

CT: computed tomography; COVID-19: coronavirus disease 2019; COPD: chronic obstructive pulmonary disease; ARDS: acute respiratory distress syndrome; HTN: hypertension; ICU: intensive care unit; WHO: World Health Organization; ECDC: European Centre for Disease Prevention and Control.

**Table 3:** Incidence of different RSNA classification features in definite and noninfected patients.

Finding	RT-PCR^∗∗^ positive (%) *n* = 22	RT-PCR negative (%) *n* = 64
*GGO* ^*∗*^		
Presence	15 (68.1)	30 (46.8)
Multiple bilateral	10 (45.4)	22 (34.3)
Multiple unilateral	2 (9)	2 (3.1)
Single unilateral	3 (13.6)	6 (9.3)
Peripheral	11 (50)	13 (20.3)
Central	2 (9)	4 (6.2)
Peripheral/central	2 (9)	13 (20.3)
Irregular	9 (40.3)	25 (39)
Round	3 (13.6)	2 (3.1)
Round/irregular	3 (13.6)	3 (4.6)

*Consolidation*		
Presence	11 (50)	22 (34.3)
Multiple bilateral	8 (36.3)	13 (20.3)
Multiple unilateral	—	—
Single unilateral	3 (13.6)	9 (14)
Peripheral	8 (36.3)	17 (26.5)
Central	2 (9)	1 (1.5)
Peripheral/central	1 (4.5)	4 (6.2)
Irregular	9 (40.9)	18 (28.1)
Round	—	—
Round/irregular	2 (9)	4 (6.2)

*Rib fracture*		
Presence	3 (13.6)	14 (21.8)
With pneumothorax	3 (13.6)	6 (9.3)
With pleural effusion	2 (9)	8 (12.5)

*Pneumothorax*		
Presence	7 (31.8)	10 (15.6)
Without rib fracture	4 (18.1)	4 (6.2)

*Hemothorax*		
Presence	11 (50)	15 (23.4)
Without rib fracture	9 (40.9)	7 (10.9)

^*∗*^GGO: ground-glass opacity. ^∗∗^RT-PCR: reverse transcription polymerase chain reaction.

**Table 4:** Diagnostic values of computed tomography findings in COVID-19 trauma patients.

Finding	Sensitivity (%)	Specificity (%)	Positive predictive value (%)	Negative predictive value (%)	Accuracy (%)	*p* value
*GGO* ^*∗*^						
Presence	68	53	33	82	56	0.05
Multiple bilateral	45	65	31	77	60	0.55
Multiple unilateral	9	96	50	75	74	0.83
Single unilateral	13	90	33	75	70	0.79
Peripheral	50	79	45	82	72	0.13
Central	9	93	33	75	72	0.9
Peripheral/central	9	79	13	71	61	0.71
Irregular	40	60	26	75	55	0.92
Round	13	96	60	76	75	0.72
Irregular/round	13	95	50	76	74	0.73

*Consolidation*						
Presence	50	65	33	79	61	0.39
Multiple bilateral	36	79	38	78	68	0.43
Single unilateral	13	85	25	74	67	0.98
Peripheral	36	73	32	77	63	0.62
Central	9	98	66	75	75	0.83
Peripheral/central	4	93	20	74	70	0.95
Irregular	40	71	33	77	63	0.51
Irregular/round	9	93	33	75	72	0.9

*Rib fracture*						
Presence	13	78	17	72	61	0.76
With pneumothorax	13	90	33	75	70	0.85
With plural effusion	9	87	20	73	67	0.89

*Pneumothorax*						
Presence	31	84	41	78	70	0.44
Without rib fracture	18	93	50	76	74	0.63

*Hemothorax*						
Presence	50	76	42	81	69	0.16
Without rib fracture	40	89	56	81	76	0.19

^*∗*^GGO: ground-glass opacity.

## Data Availability

SPSS data of the participants are available upon reasonable request from the corresponding author.

## Abbreviations

CT: Computed tomography
ICU: Intensive care unit
COVID-19: Coronavirus disease 2019
COPD: Chronic obstructive pulmonary disease
RSNA: Radiological Society of North America
WHO: World Health Organization
RT-PCR: Reverse transcription polymerase chain reaction
GGO: Ground-glass opacity
SIRS: Systemic inflammatory response syndrome
NLR: Lymphocyte-to-neutrophil ratio
ISS: Injury severity score
AIS: Abbreviated injury score.
